# Development of Bioactive Niobium Oxalate-Based Desensitizer: Permeability and Formation of Nanoprecursors

**DOI:** 10.1590/0103-644020256534

**Published:** 2025-11-21

**Authors:** Luanna Marinho Sereno Nery Saldanha, Paulo Vitor Campos Ferreira, Felipe Silva Gomes, Gabriel Nima Bermejo, Clenilton Costa Dos Santos, José Bauer, Darlon Lima Martins

**Affiliations:** 1 Dentistry Biomaterials Laboratory (Biomma), Postgraduate Program in Dentistry at the Federal University of Maranhão (PPGO-UFMA), Zip Code 65080-805 São Luís, MA, Brazil; 2 Department of Biomedical Sciences, Ethics, Research, and Education School of Dentistry, Universidad de los Andes, Santiago, Chile; 3 Graduate Program in Physics, Federal University of Maranhão (UFMA), São Luís, 65085-580, Brazil

**Keywords:** Bioactive, Dentin permeability, Oxalates, Desensitizers, Dentin hypersensitivity

## Abstract

To evaluate the obliteration of dentinal tubules by experimental pastes with different concentrations of niobium oxalate compared to commercial pastes. Experimental pastes were synthesized from mixtures of different concentrations of sodium carboxymethylcellulose and ammonium niobium oxalate. Twenty-five dentin discs, each 1.5 mm thick, were obtained from molars and divided into five experimental groups (n=5): 1) Oxagel (Kota, Brazil); 2) Desensibilize KF 2% (FGM, Brazil); 3) Paste with 5% niobium oxalate (wt%); 4) Paste with 10% niobium oxalate; 5) Paste with 20% niobium oxalate. The pastes were applied, and initial permeability readings were taken immediately, as well as at 7, 14, and 21 days. Measurements were made using the THDO3d device (Odeme, Brazil). Scanning electron microscopy images and analysis of precipitate formation were performed after 21 days (SEM/EDS). The commercial groups showed a greater reduction in Lp (hydraulic conductance) after 7 days, which remained constant until 21 days. The experimental groups, regardless of concentration, showed a high reduction in permeability immediately. SEM images revealed the formation of a thick layer composed of precipitate and the successive application of the material for all experimental groups. Niobium oxalate pastes are capable of significantly reducing dentinal permeability in both the short and long term, regardless of the concentration applied.

## Resumo

O presente estudo se propõe a avaliar a obliteração dos túbulos dentinários por pastas experimentais com diferentes concentrações de oxalato de nióbio em comparação com pastas comerciais. Pastas experimentais foram sintetizadas a partir de misturas de diferentes concentrações de carboximetilcelulose de sódio e oxalato de nióbio de amônio. Vinte e cinco discos de dentina, cada um com 1,5 mm de espessura, foram obtidos de molares e divididos em 5 grupos experimentais (n=5): 1) Oxagel (Kota, Brasil); 2) Desensibilize KF 2% (FGM, Brasil); 3) Pasta com 5% de oxalato de nióbio (wt%); 4) Pasta com 10% de oxalato de nióbio; 5) Pasta com 20% de oxalato de nióbio. As pastas foram aplicadas e leituras iniciais de permeabilidade foram feitas imediatamente, e aos 7, 14 e 21 dias. As medições foram feitas utilizando o dispositivo THDO3d (Odeme, Brasil). Imagens de microscopia eletrônica de varredura e análise da formação de precipitados foram realizadas após o período de 21 dias (MEV/EDS). Os grupos comerciais mostraram uma maior redução em Lp após 7 dias, que permaneceu constante até 21 dias. Os grupos experimentais, independentemente da concentração, mostraram uma alta redução na permeabilidade imediatamente. As imagens de MEV mostraram a formação de uma camada espessa composta pela formação de precipitados e sucessivas aplicações do material para todos os grupos experimentais. Pastas de oxalato de nióbio são capazes de reduzir significativamente a permeabilidade dentinária tanto a curto quanto a longo prazo, independentemente da concentração aplicada.

**Figure:**
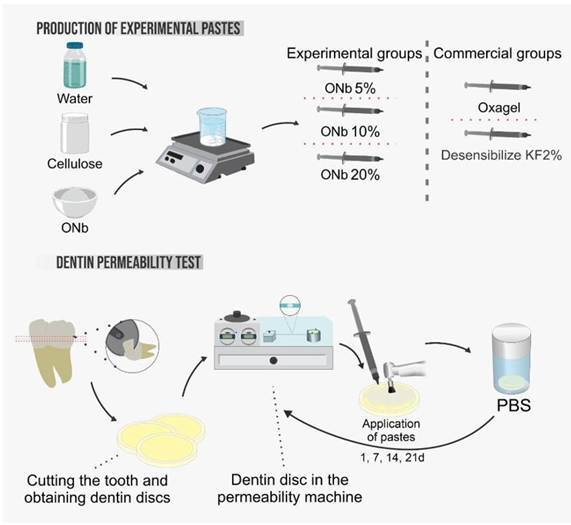

## Introduction

Dentin hypersensitivity (DH) is a clinical-pathological condition caused by the exposure of a region of dentin and the consequent opening of the dentinal tubules. The prevalence of this condition has increased worldwide and is associated with various etiologies ^1^ that can act independently or in a multifactorial manner. Among the primary etiological factors of dentin hypersensitivity, abrasion, erosion, and attrition, caused, respectively, by mechanical wear, chemical dissolution, and tooth-to-tooth contact, are of paramount importance, as they progressively degrade dental enamel, expose dentinal tubules to the oral environment, and elicit hypersensitivity ^2^. Treatment strategies for DH involve two mechanisms of action: reducing nerve excitability by depolarizing cells with potassium salts that alter the electrical potential, and tubule obliteration, which can occur through the use of fluorides, oxalates, arginine, polymers, among others ^3^. In the long term, tubule occlusion is the most effective way to reduce the impacts caused by DH ^4^. The most common mechanism for tubule obliteration consists of the deposition of inorganic precipitates from oxalate and fluoride salts ^5^.

The application of potassium oxalate is one of the oldest forms of DH treatment. The first in vitro studies date back to the late 1970s and early 1980s ^5^. The mechanism of action initially involves the dissociation of oxalate groups and K ions when the compound is in contact with an aqueous medium, followed by the formation of calcium oxalate crystals. This process occurs almost instantaneously on the dentin surface. The crystals formed consist of dihydrated calcium oxalate and are formed from calcium ions derived from the smear layer and dentinal fluid ^5^. The crystals formed are relatively insoluble in acidic conditions and thus resistant to dissolution by acidic foods and beverages ^6^.

Although the literature reports good performance of potassium oxalate in the treatment of DH, its clinical effectiveness may be considered limited due to factors such as susceptibility to dissolution in the oral environment, temporary dentinal tubule occlusion, and, consequently, lack of long-lasting effects. Materials such as hydroxyapatite, bioactive glasses, silica nanoparticles, amorphous calcium phosphate, and others have been applied in in vitro studies involving DH ^4^ ^,^ ^7^ ^,^ ^8^. These biomaterials aim to interact with body fluids and induce the formation of carbonated hydroxyapatite and/or precursor mineral phase within collagen fibrils and dentinal tubules, remineralizing and occluding this region ^9^.

The mechanism for this mineral-formation induction process involves heterogeneous nucleation ^10^. This phenomenon occurs through the ionic dissolution of bioactive particles and the saturation of the medium, as well as at the particle surface due to the presence of specific functional groups such as Si-OH, Ti-OH, Zr-OH, Ta-OH, and Nb-OH ^10^ ^,^ ^11^. Thus, various types of biomaterials have been developed that incorporate these elements.

Among these elements, niobium has been reported to have high biocompatibility, capable of inducing mineral formation and stimulating cell proliferation/differentiation ^11^ ^,^ ^12^ ^,^ ^13^. Miyazaki et al. (2001) demonstrated that the formation of apatite crystals on the surface of Nb oxide synthesized via the sol-gel process, immersed in mineralizing solution, is directly associated with the presence of Nb-OH functional groups ^11^. Recently, various dental materials have been developed by incorporating bioactive particles containing Nb ions, thereby imparting bioactive properties to these materials ^14^ ^,^ ^15^ ^,^ ^16^.

To evaluate the effectiveness of niobium oxalate in promoting dentinal tubule occlusion, experimental pastes containing varying concentrations of this compound were developed. These pastes are designed to act through two complementary mechanisms: the deposition of oxalate crystals and the nucleation of apatite, thereby enhancing the sealing of exposed tubules. Accordingly, this study aimed to compare the occlusive potential of the experimental formulations with that of a control group treated with a conventional desensitizing agent, monohydrated potassium oxalate. It is hypothesized that the niobium oxalate-based pastes will demonstrate superior efficacy in dentinal tubule occlusion compared to the standard treatment.

## Materials and methods

### Design Experimental

This *in vitro* study was divided into two main phases: the production of experimental pastes and the dentin permeability test. Human third molars were used, following approval from the Research Ethics Committee of the Federal University of Maranhão (CAAE - 63874622.3.0000.5087). In the first phase, the experimental pastes were produced by combining water, cellulose, and ONb (Niobium Oxalate) in concentrations of 5%, 10%, and 20%. These experimental pastes were compared with two commercial groups, Oxagel and Desensibilize KF2%.

In the second phase, teeth were cut to obtain dentin discs, which were then positioned in a dentin permeability machine. The pastes were applied to the dentin discs at intervals of 1, 7, 14, and 21 days, with permeability being evaluated after each application (Graphic Abstract).

### Obtaining bioactive experimental pastes

To produce the pastes, sodium carboxymethylcellulose salt (Sigma-Aldrich, Haverhill, Mass, USA) and ammonium niobium oxalate were used. (NH_4_NbO(C_2_O_4_)_2_) (Sigma-Aldrich, Haverhill, Mass, USA). Homogenized with a magnetic stirrer. The composition of the experimental and commercial pastes is shown in Table 1.

**Table 1:** Composition of the experimental and commercial pastes used in this study.

Groups	Active compounds	Other ingredients
OXAGEL (Kota, Brazil)	Monohydrated Potassium Oxalate	Carboxymethylcellulose, distilled water, chlorhexidine, fluoride.
Desensibilize KF2% (FGM, Brazil)	5% Potassium Nitrate and 2% Sodium Fluoride	Distilled water, glycerin, neutralizing agent, and thickener.
ONb 5% ONb 10% ONb 20%	5 to 20% ammoniacal niobium oxalate (NH_4_NbO(C_2_O_4_)_2_)	2-5% sodium carboxymethyl cellulose salt, water.

### Preparation of test specimens

Twenty-five human third molars were used in this study. Dentin crown segments were obtained by first removing the roots 1.0 mm beneath the cementum-enamel junction (CEJ) using a pre-fabricated 1.0 mm acrylic spacer to guide the cut, followed by sectioning with a slow-speed water-cooled diamond saw (Isomet 1000, Buehler, USA). The occlusal enamel was subsequently removed with a parallel cut to expose the deep dentin. Pulpal tissue was carefully removed from the exposed pulp chamber. The remaining dentin thickness was standardized to 1.5 mm and confirmed using a digital caliper (Mitutoyo, Japan). Twenty-five dentin discs were then divided into five groups (n = 5) ^9^. Next, the smear layer was standardized using 600-grit sandpaper for 30 seconds; the discs were subsequently inspected under a stereomicroscope (Kozo Optical and Electronic Instrumental, Nanjing, Jiangsu, China) at 40× magnification to ensure the absence of enamel on their surface.

### Initial permeability definition and sample randomization

For the randomized division of the specimens among the treatment groups (Table 1), the initial permeability (Lp initial) was determined. Based on the measurement results, the specimens were distributed so that each group had specimens with the same permeability pattern, thereby ensuring group homogeneity.

In this Lp initial analysis, the specimens were evaluated for their maximum permeability. Before analysis, dentin discs were conditioned with 37% phosphoric acid for 30 seconds (Attaque Gel, Biodinâmica, Brazil), washed with running water for the same duration, dried with absorbent paper, and then placed in the permeability machine. Measurements were made according to the manufacturer's manual for the THDO3d dentin permeability measurement device (Odeme Ltda, Joaçaba, SC, Brazil).

### Specimen treatment

Each group was treated with the pastes as defined in Table 1. For all groups, the pastes were applied to the dentin disc surface using a Robson brush at low speed for 10 seconds and left to rest for 10 minutes. The process was then repeated, with reactivation for 10 seconds. Excess material was removed with a damp cotton ball, and dentin permeability was measured.

The treatment was performed after reading the Lp initial, then determining the Lp immediate, and after each reading (7, 14, and 21 days). After applying the pastes, the specimens were stored in a phosphate-buffered saline (PBS) solution in an incubator at 37°C (502C; FANEM, SP, Brazil).

### Dentin permeability

For the dentin permeability test, a split chamber model was used, in which the pulpal side of the dentin disc is placed in contact with a pressurized fluid, and the other side is in contact with the environment at atmospheric pressure.

During the test, the perfusion fluid (distilled water) exits the reservoir under controlled pressure (±20 mmHg) and passes through a capillary tube into the perfusion chamber, filling the entire system. An air bubble is introduced into the capillary tube via a secondary valve. The displacement of this bubble assesses the rate of fluid movement through the dentin discs. In this study, five readings of the bubble's linear displacement were taken for each specimen over a 2-minute period, using a digital caliper with a resolution of 0.01 mm.

Permeability calculations were performed using Odeme Analysis data analysis software (Joaçaba, SC, Brazil). Dentin permeability was expressed in terms of hydraulic conductance (Lp) using the following equations:

$$LP=\frac{Q}{P*Asde}$$

e

$$Q=\;\frac{Vp*D}{L*T}$$

Where:

Lp Hydraulic conductance expressed in (µL/min.cm H2O.cm²)

Q Filtration rate (µL/min) - Obtained by the second equation

P Hydrostatic pressure difference across the dentin (cm H2O)

Asde Exposed dentin surface area (cm²);

Vp Standardized volume (µL);

D Bubble displacement in the capillary tube (mm);

L Length of the capillary tube (mm);

T Test duration (min).

The bubble displacement (D) and hydrostatic pressure (P) were given variables, while the others were constant.

### Surface analysis and mineral precipitate formation

Three samples from each group were selected after the end of the dentin permeability test and taken to the SEM/EDS (TM3030, Hitachi, Tokyo, Japan), where magnifications of 100x, 500x, and 1000x were obtained for morphological characterization of the precipitates deposited on the surface. Raman analysis of the dentin surface was performed using a Horiba-Jobin-Yvon triple spectrometer (model T64000) equipped with a confocal system, liquid-N_2_ cooling, and a solid-state laser (LAS-532-100-HREV, Kyoto, Japan). A wavelength of 532.0 nm at 14 mW was utilized. The samples were irradiated by the laser, and adjustments were made using an Olympus microscope (Tokyo, Japan) with an MPLN 100X objective lens. The analysis was conducted at various points on the surface, including along the vertical direction.

### Statistical analysis

Statistical analysis was performed using SigmaPlot software (Systat Software Inc., San Jose, California, USA). Data normality was assessed using the Shapiro-Wilk test (p > 0.05), and equality of variances was evaluated with the Brown-Forsythe test (p > 0.05). Dentin permeability data were subjected to a two-way repeated-measures analysis of variance (ANOVA) with material and time as factors, followed by Holm-Sidak post-hoc tests (α = 0.05).

## Results

### Dentin permeability

The assumption of normality was confirmed using the Shapiro-Wilk test (P = 0.751), while the assumption of homoscedasticity was verified via the Brown-Forsythe test (P = 0.365). The statistical power of the performed tests, considering an alpha level of 0.05, was 1.000.

**Figure 1:**
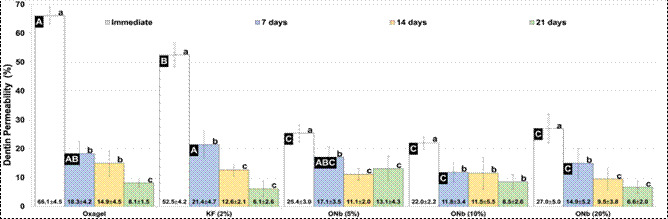
Graph of reduction in dentin permeability of the different experimental groups of niobium oxalate (5, 10 and 20%) and commercial pastes (Oxagel and KF 2%) in the immediate periods, 7, 14 and 21 days*.

The dentin permeability results of commercial and experimental pastes tested at different times are shown in Figure 1. The analysis of variance showed an interaction between the main factors (p < 0.001). It is noted that the groups treated with the commercial pastes Oxagel and KF 2% had a low impact on reducing dentin permeability immediately after the first application (immediate). The experimental groups treated with niobium oxalate pastes showed a higher reduction in dentin permeability immediately after the first application compared to commercial pastes (p < 0.001). The experimental and commercial groups showed no statistically significant difference in permeability values at periods of 14 and 21 days (p > 0.05).

The analysis of variance showed an interaction between the main factors (p < 0.001). It is noted that the groups treated with the commercial pastes Oxagel and KF 2% had a low impact on reducing dentin permeability immediately after the first application (immediate). The experimental groups treated with niobium oxalate pastes exhibited a significant reduction in dentin permeability immediately after the first application, compared to commercial pastes (p < 0.001). The experimental and commercial groups showed no statistically significant differences in permeability values at 14 and 21 days (p > 0.05).

### Surface analysis and mineral precipitate formation

**Figure 2:**
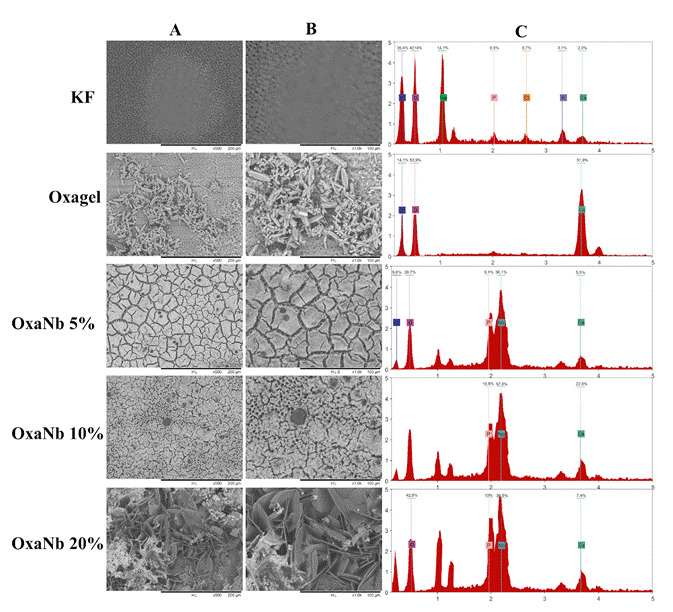
Scanning electron microscopy images: (A): 500X magnification and (B): 1000X magnification of the precipitates formed on the surfaces of the dentin specimens (C): Analysis of the composition spectra (EDS) of the precipitates formed on the dentin surface.

**Figure 3:**
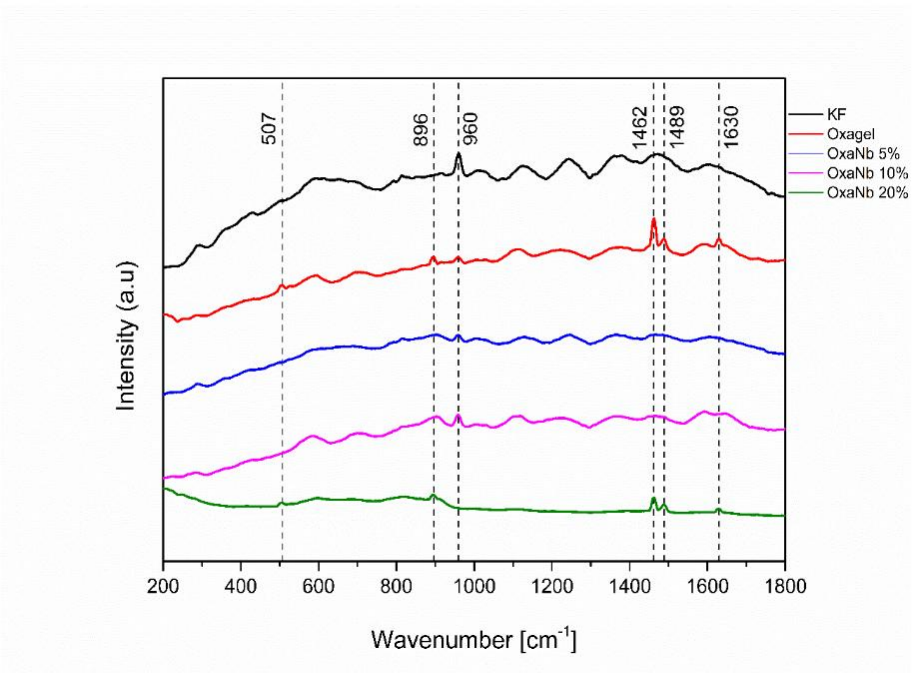
Raman spectra of dentin surfaces treated with different experimental pastes: Desensibilize KF2% (black), Oxagel (red), OxaNb 5% (blue), OxaNb 10% (magenta), and OxaNb 20% (green). Peaks at 507, 896, 1462, 1489, and 1630 cm⁻¹ indicate the presence of calcium oxalate crystals. The peak at 960 cm⁻¹, absent in OxaNb 20%, indicates the presence of hydroxyapatite crystals.

Samples from all experimental groups showed precipitate formation, except those treated with Desensibilize KF2%. Dentin surfaces treated with Oxagel exhibited the formation of fine, elongated crystals, resembling bipyramidal prisms (Figure 2A and B). EDS analysis performed on these structures indicated the presence of O, C, and Ca elements (Figure 2C). Additionally, Raman spectra obtained in the same region demonstrated the presence of peaks at 507, 896, 1462, 1489, and 1630, related to the formation of calcium oxalate crystals (Figure 3).

The samples treated with experimental pastes containing different concentrations exhibited a typical pattern in precipitate formation: a thick layer deposited on the surface of the dentin samples (Figure 2). Cracks were observed in this layer after the drying process, which is pertinent to scanning electron microscopy analysis. Additionally, specimens from the OxaNb 20% experimental group showed the formation of calcium oxalate crystals, as demonstrated by the Raman and EDS spectra (Figures 2 and 4). However, SEM images revealed precipitates with a morphology distinct from the crystals observed in the Oxagel group samples (Figure 2). Furthermore, EDS data indicated the presence of Nb and P ions, in addition to the elements associated with calcium oxalate crystals (O, C, and Ca). In the other groups, OxaNb 5% and 10%, the precipitates appeared as a thick layer deposited on the dentin, composed of elements such as Nb, Ca, and P (Figure 2).

The presence of the 960 peak was observed in the Raman (Figure 3) spectrogram of all evaluated specimens, except for those treated with the OxaNb 20% experimental paste. This peak characterizes the presence of hydroxyapatite crystals, which are a component of the dentin composition.

## Discussion

Oxalate salts have a high capacity to obliterate dentinal tubules and, for this reason, have been used in the treatment of dentin hypersensitivity since 1975 ^17^. These salts, when solubilized, have an acidic pH capable of removing the smear layer and interacting with the dentin surface ^5^. Ionized oxalic groups, when in contact with dentinal fluid, deposit a layer of calcium oxalate crystals responsible for obliterating the tubules ^18^. Based on the successful application of oxalate salts in the treatment of HD, as well as the promising application of niobium-based biomaterials, this study proposes the unprecedented application of ammonium niobium oxalate salt as an effective alternative in tubule obliteration.

In addition to the short-term effectiveness of obliteration, this study evaluated the behavior of the materials over 21 days. Commercial pastes showed a low reduction in dentin permeability values immediately after the first application. These values can be attributed to the low amount of bioactive materials and the short period of interaction between the material and the dentin surface, which results in the formation of insufficient precipitates to obliterate the dentin effectively. In the present study, a niobium oxalate salt was used, which can generate chemically unstable and acidic precipitates. The literature indicates that pH is a crucial factor in the precipitation process. The optimal pH for the formation of stoichiometric hydroxyapatite ranges between 8 and 10.

In contrast, the acidic character of niobium oxalate may not provide ideal conditions for the formation of chemically stable precipitates. Furthermore, studies using bioactive glasses and mineral-rich particles (e.g., 45S5 and niobophosphate) have demonstrated a superior capacity to form robust precipitate layers that effectively occlude dentinal tubules ^19^. Another important consideration is the storage medium; while our study used PBS, other investigations employ supersaturated SBF solutions, which may enhance precipitate formation and stability ^20^. These factors, combined, may explain the limited tubular occlusion observed in our results.

The efficiency in reducing the permeability of commercial pastes is evident after 7 days. On the other hand, dentin surfaces treated with experimental pastes containing niobium oxalate showed an immediate reduction in dentin permeability, regardless of the concentration (Figure 1).

The explanation for the good results obtained by pastes containing niobium is based on their mechanism of action, which differs from the traditional phenomenon attributed to pastes containing oxalate salts and other products used (fluorides, arginine, hydroxyapatite, among others). The mechanism of obliteration of dentinal tubules by niobium oxalate combines two strategies: 1) formation of calcium oxalate crystals from oxalic groups from the dissolution of the paste and Ca^++^ ions from the oral environment, as observed in the Raman spectroscopy analyses (Figure 3); 2) Induction of the formation of a layer of hydroxyapatite-type precipitates from Nb-OH ions resulting from the dissolution of niobium oxalate salt, as observed in the SEM/EDS analyses (Figure 2) ^21^.

In this context, the nucleation process is influenced by various factors, including the surface energy of the particle used and its chemical composition, which are responsible for ionic interactions and, consequently, the formation of precipitates on the dentin surface and within the dentinal tubule openings. Surface functional groups, such as hydroxyl and phosphate groups, serve as nucleation sites, facilitating the deposition of minerals. Furthermore, environmental factors, including pH, ionic concentration, and exposure time, critically affect the stability and growth of these precipitates. Therefore, these mechanisms collectively contribute to the effectiveness of bioactive materials in clinical applications such as remineralization and dentin obliteration, as demonstrated in previous studies ^22^ ^,^ ^23^ ^,^ ^24^.

Studies have shown the use of niobium in the production of bioceramic materials, phosphate glasses, and hydroxyapatite particles ^24^ ^,^ ^25^. This occurs due to the low level of cytotoxicity in the different forms of presentation (oxide, chloride, oxalate, etc.), as well as attributing benefits to the properties of these materials, such as crystallinity, morphology, thermal stability, and dissolution rate in physiological conditions ^25^. Amorphous and crystalline phases exhibit different behaviors regarding their capacity to form precipitates. A bioactive particle in an amorphous phase may enhance the potential for the formation of occlusive precipitates on dentin. Regarding morphology, two predominant forms are frequently observed: needle-like structures and circular agglomerates. The latter, due to their shape and size, are more likely to penetrate deeply into the dentinal tubules, contributing more effectively to their sealing. Thermal stability and dissolution rate also play critical roles, promoting controlled dissolution and, consequently, the gradual release of ions. Together, these properties-along with other variables that are often unpredictable, such as the local environment, the composition of the storage solution, and the application conditions-govern the precipitation behavior and ultimately determine the degree of tubule occlusion achieved.

Karlinsey et al. (2016) demonstrated the ability of self-assembled niobium oxide crystalline microstructures to act as an efficient nucleating agent for hydroxyapatite and other Ca-P minerals, after immersion in a supersaturated artificial saliva solution ^12^. Miyazaki T et al. relate the ability to form apatite to the presence of Nb-OH groups, which act as a nucleation site ^11^.

The low permeability values were maintained in the other periods of analysis, in a similar behavior to the other experimental groups. The different concentrations of niobium oxalate (5, 10, and 20%) used showed the same behavior and similar values. For the experimental group, it is possible to observe in the SEM images the deposition of a thick layer of precipitate rich in calcium and niobium. This layer may be associated with the deposition of precipitates due to successive applications of the material.

This current study is innovative in suggesting the use of a niobium oxalate-based paste as a desensitizer for human dentin. The results are considered promising, given the high potential for reducing permeability immediately after application, in addition to maintaining tubule obliteration until day 21. However, clinical tests must be conducted to confirm this efficiency in treating hypersensitivity.

## Conclusion

The experimental niobium oxalate pastes resulted in a considerable reduction in dentin permeability after the first application, as well as throughout the treatment period. However, in the periods of 14 and 21 days, all materials used presented low dentin permeability values.
